# Colon cancer cells evade drug action by enhancing drug metabolism

**DOI:** 10.1038/s41388-025-03472-3

**Published:** 2025-07-10

**Authors:** Bojie Cong, Teena Thakur, Alejandro Huerta Uribe, Evangelia Stamou, Sindhura Gopinath, Owen Sansom, Oliver Maddocks, Ross Cagan

**Affiliations:** 1 School of Cancer Sciences, University of Glasgow, Wolfson Wohl Cancer Research Centre; Garscube Estate, Switchback Road, Bearsden, Glasgow, Scotland UK; 2 CRUK Scotland Institute, Garscube Estate, Glasgow, Scotland UK; 3 Department of Cell, Developmental and Regenerative Biology, Icahn School of Medicine at Mount Sinai, New York, NY USA; 4 Faeth Therapeutics Inc, Austin, TX USA

**Keywords:** Cancer therapy, Cancer genetics

## Abstract

Colorectal cancer (CRC) is the second leading cause of cancer deaths worldwide. One key reason is the lack of durable therapies that target KRAS-dependent disease, which represents approximately 40% of CRC cases. Here, we use liquid chromatography/mass spectrometry (LC/MS) analyses on *Drosophila* CRC tumour models to identify multiple metabolites in the glucuronidation pathway—a toxin clearance pathway that impacts most drugs—as upregulated in trametinib-resistant *RAS/APC/P53* (“*RAP*”) tumours compared to trametinib-sensitive *Ras*^*G12V*^ single mutant tumours. Genetic inhibition of different steps along the glucuronidation pathway strongly reversed *RAP* resistance to trametinib; conversely, elevating glucuronidation pathway activity was sufficient to direct trametinib resistance in *Ras*^*G12V*^ animals. Mechanistically, pairing oncogenic RAS with hyperactive WNT activity strongly elevated PI3K/AKT/GLUT signalling, which in turn directed elevated glucose uptake and glucuronidation; our data also implicate the pentose phosphate pathway in this process. We provide evidence that this mechanism of trametinib resistance is conserved in a *KRAS/APC/TP53* mouse CRC tumour organoid model. Finally, we identify two clinically accessible approaches to inhibiting drug glucuronidation: (i) blocking an initial HDAC1-mediated deacetylation step of trametinib with the FDA-approved drug vorinostat; (ii) reducing blood glucose by the alpha-glucosidase inhibitor acarbose. Overall, our observations demonstrate a key mechanism by which oncogenic RAS/WNT activity promotes increased drug clearance in CRC and provides a practical path towards abrogating drug resistance in CRC tumours.

## Introduction

Despite recent advances in RAS pathway therapies, RAS-mutant colorectal cancer (CRC) has proven poorly sensitive to most targeted CRC therapies in the clinics [1]. This is somewhat surprising, as RAS pathway inhibitors have shown strong efficacy in *RAS*-mutant CRC pre-clinical studies. One important factor that has emerged is the role of genetic complexity: drug resistance typically increases in preclinical models that are more genetically complex [2, 3]. We recently reported this phenomenon in Drosophila CRC models, both preclinically and in complex fly avatars as a part of a clinical trial: compared to oncogenic RAS alone, additionally targeting tumour suppressors APC and P53 (“RAP”) consistently led to emergent drug resistance [3, 4]. However, the mechanisms that link genetic complexity to resistance across a broad spectrum of targeted therapies remain poorly understood.

A growing number of studies have shown that genetic and signalling complexity can strongly influence drug response. For example, genomic mutations that lead to amplification or ‘rewiring’ of key signalling pathways have been linked to failure of targeted therapies [5, 6]; however, co-targeting of these pathways has to date still failed to yield durable KRAS-mutant CRC treatments [7–10]. An alternative possibility is emergence of a drug target-agnostic mechanism in response to genomic complexity. *KRAS*, *APC* and *TP53* are the most commonly mutated genes reported for human CRC [3, 11]: adenoma progression is associated with loss of *APC* paired with oncogenic mutations in *KRAS*; malignant transformation is associated with additional mutations in *TP53* [12, 13]. We therefore focused on this canonical multi-gene mutation profile commonly observed in human CRC tumours.

Oncogenic RAS is a key driver for tumour progression in up to 25% of all human cancers [14]. As such, components of the RAS/MAPK pathway remain a high priority for targeted therapy. For example, the Food and Drug Administration (FDA) approved MEK inhibitor trametinib proved effective in preclinical CRC models, but showed no therapeutic benefit in CRC patients [15, 16]. Here, we report an LC/MS analysis comparing drug response in Drosophila *Ras*^*G12V*^ (trametinib sensitive) and *RAS-APC-P53* (*RAP*; trametinib resistant) hindgut tumours. We found that drug resistance in *RAP* tumours was associated with upregulated drug metabolism via glucuronidation, a primary toxin clearance pathway used by cells to clear most cancer drugs. Blocking this upregulation had no direct effect on hindgut tumours but restored drug sensitivity in *RAP* Drosophila and mouse organoid tumours to a level that mirrored tumours with *Ras*^*G12V*^ alone. We further demonstrate that patient-accessible drugs such as vorinostat, predicted to block a key pre-glucuronidation step of trametinib, strongly sensitized tumours to trametinib. Together, our results demonstrate how a canonical CRC mutation profile elevates a key detoxification pathway to promote general drug resistance; interfering with this process provides a blueprint for sensitizing genetically complex tumours to targeted therapies.

## Materials and methods

### Drosophila strains and genetics

Fly lines were cultured at room temperature or 25–29 °C on standard fly food or food-plus-compound. Fly food contained tayo agar 10 g, soya flour 5 g, sucrose 15 g, glucose 33 g, maize meal 15 g, wheat germ 10 g, treacle molasses 30 g, yeast 35 g, nipagin 10 ml, propionic acid 5 ml in 1000 ml water. Transgenes used (Bloomington Drosophila Stock Center number): *byn-gal4* (hindgut-specific line, V. Hartenstein), *UAS-Ras*^*G12V*^ (second chromosome, G. Halder), *tub-gal80*^*TS*^ (BDSC_7017), *w*^*1118*^ (BDSC_3605), *UAS-mCD8-GFP* (BDSC_5137), *UAS-Hex-C-RNAi* (BDSC_57404), *UAS-UGP-RNAi* (BDSC_50902), *UAS-GlcAT-P-RNAi* (BDSC_67771), *UAS-sgl-RNAi* (BDSC_65348), *UAS-Akt-RNAi* (BDSC_82957), *UAS-plx-RNAi* (AS160, BDSC_66313), UAS-HDAC1-RNAi (BDSC_36800), and UAS-Arm^S10^ (Arm^CA^, BDSC_4782), UAS-pgd-RNAi (BDSC_65078), UAS-zw-RNAi (BDSC_50667), UAS-rpi-RNAi (BDSC_67847), UAS-nej-RNAi (BDSC_37489).

### Construction of the Drosophila RAP model

As previously described [4], a pWalium expression vector was engineered with three Multiple Cloning Sites (MCS) downstream of UAS responsive elements. The RAP model was designed as a single plasmid construct incorporating the following: (i) oncogenic mutant Ras85D^G12V^ in the first multiple cloning site (MCS), (ii) 4 short 21 bp hairpins targeted to downregulate Apc plus 4 to downregulate P53 as a single 8-mer hairpin cluster into the third MCS with micro-RNA and intron derived spacers and loop sequences as previously described [4]. The resulting plasmid was then stably inserted into the 2^nd^ chromosome attP40 genome ‘landing site’. The sequence for the P53-Apc 8-mer:

1 actctgaata gggaattggg aattgagatc tgttctagac catattcagc ctttgagagt tggacgttca gttcaagtct atagttatat tcaagcatat

101agacttgaac tgaacgtcca gcgaaatctg gcgagacatc gagtagtgcc accaaaagtt agccgcgttg tggaaaatcc ccatattcag cctttgagag

201 tcaacgtgga cgttcagttc aatagttata ttcaagcata ttgaactgaa cgtccacgtt ggcgaaatct ggcgagacat cggagggaaa tggagaacgc

301 aaaaatccca ttataatgga accatattca gcctttgaga gtccggatga acaaggcctt caatagttat attcaagcat attgaaggcc ttgttcatcc

401 gggcgaaatc tggcgagaca tcgatgtgct tgatcgtaac tccatccaaa ctcgatatta acccatattc agcctttgag agttcggtgg ttattgcttc

501 agcatagtta tattcaagca tatgctgaag caataaccac cgagcgaaat ctggcgagac atcgacaaat aatgttgcaa taaccagttg aaaccaatgg

601 aatccatatt cagcctttga gagtctcaaa gttgtgcaac tcttatagtt atattcaagc atataagagt tgcacaactt tgaggcgaaa tctggcgaga

701catcgaacta acccgttcac ctgcgacaat ttttaatcta ttttccatat tcagcctttg agagtctgga cgaccagctt cgatgatagt tatattcaag

801 catatcatcg aagctggtcg tccaggcgaa atctggcgag acatcgagac cacgatcgaa agaggaaaaa cggaaaacga acgaaccata ttcagccttt

901 gagagtaaag atggacaaga agtacgatag ttatattcaa gcatatcgta cttcttgtcc atctttgcga aatctggcga gacatcggga ctagttttca

1001 ttatttatca gccagcacca acaacaccat attcagcctt tgagagtgca gctaaagatg gacaagaata gttatattca agcatattct tgtccatctt

1101 tagctgcgcg aaatctggcg agacatcgtt ggtactcgag atagtttgta tgaaatattt atatttttag cggccgcaag aa

### Chemicals

Drugs and compounds were as follows: trametinib (Selleckchem_S2673 or biorbyt), UDP-glucose (Abcam_ab120384), sucrose (Sigma-Aldrich_S0389), LY294002 (Selleckchem_S1105), vorinostat (Selleckchem_S1047), phenacetin (Selleckchem_S2577), binimetinib (Selleckchem_S7007), selumetinib (Selleckchem_S1008), acarbose (Selleckchem_S1271), D-ribulose (Sigma-Aldrich_83899), fasentin (Sigma-Aldrich_F5557) and alpelisib (Selleckchem_S2814). Drug and compound stocks were diluted in Dimethylsulfoxide (DMSO) or water; drugs were then mixed into standard fly food with final DMSO concentration 0.1% to prevent toxicity.

### Liquid chromatography–mass spectrometry (LC–MS) in *Drosophila*

Third instar larvae were dissected in 1× PBS, and hindguts (n = 30) were collected and lysed on ice in 200 µl of extraction buffer (methanol:acetonitrile:water volume ratio 50:30:20). Tissue was homogenised and centrifuged at 13,000 rpm for 15 min at 4 °C. Supernatants were transferred to clean 1.5 ml tubes and stored at –80 °C. Metabolites were profiled using liquid chromatography–mass spectrometry (LC-MS) with an Accela 600 LC system coupled to an Exactive Orbitrap mass spectrometer (Thermo Scientific). Chromatographic separation was performed using a Sequant ZIC-pHILIC column (4.6 × 150 mm, 3.5 µm particle size; Merck). The mobile phase consisted of solvent A (20 mM ammonium carbonate in water) and solvent B (acetonitrile). The gradient was programmed to increase from 20% to 80% A over 30 minutes, followed by a 5-min wash at 92% A and re-equilibration for 10 minutes at the initial 20% A. Total run duration was 45 min. The mass spectrometer was operated in full scan mode with polarity switching across a 70–1200 m/z range and a resolving power of 50,000. Raw LC-MS files were converted to mzML format using MSConvert (ProteoWizard, RRID:SCR_012056), then processed in MZMine 2.3 for peak detection. Metabolites were identified using an in-house spectral library. Metabolites were assigned to known pathways with functional and enrichment analyses in MetaboAnalyst 5.0 (RRID:SCR_015539) [17].

### Statistical analysis

Eggs were collected for 24 h in drug-containing food at 18 °C to minimize transgene expression during embryogenesis to prevent embryonic effects or lethality. After 3 days, tubes were transferred to the appropriate temperature to induce transgene expression; the number of surviving *Drosophila* adults including male, and female was quantified after 2 weeks. Drawing from our previously published experience, all experiments were repeated three times with a total number of samples exceeding 5. Statistical analysis was performed using Prism9, and statistical tests were conducted using the Mann–Whitney test or ordinary one-way ANOVA. N.S *P*( > 0.12), *P(0.033), **P(0.002), ***P(0.001), and ****P( < 0.0001). *P*-values ≤ 0.033 were considered significant. All statistical data were summarized in Supplementary Table S2. All detailed genotypes were summarized in Supplementary Table S3.

### Western blot analysis of *Drosophila* hindguts

Third instar larvae were dissected in 1x PBS and hindguts (30) were put into 2x Laemmli sample buffer (2.1% SDS, 26.3% glycerol, 65.8 mM Tris-HCl (pH 6.8), 0.01% bromophenol blue, 5% 2-mercaptoethanol, BIO-RAD_1610747). After homogenization at 100 °C for 5 min, samples were centrifuged at 13,000 rpm for 10 min, and lysate supernatants were used for western blot analysis. For signal detection, SuperSignal West Pico PLUS Chemiluminescent Substrate (Thermo Fisher Scientific_34580) was used. Primary antibodies used were as follows: anti-Phospho-Akt (Cell Signaling Technology_4060, 1:500 in 5%BSA/TBST (0.1% Tween20 in TBS)), anti-Akt (Cell Signaling Technology_4691, 1:1000 in 5%BSA/TBST), monoclonal anti-α-Tubulin (Sigma-Aldrich_T5168-2ML, 1:5000 in 5%BSA/TBST). Secondary antibodies used: anti-mouse IgG, HRP-linked antibody (1:2000 in 5%BSA/TBST, Cell Signaling Technology_7076S), anti-rabbit IgG, and HRP-linked antibody (1:2000 in 5%BSA/TBST, Cell Signaling Technology_7074S).

### Endogenous UDP release assay, glucose assay, and HDAC activity assay

Third instar larvae were dissected in 1x PBS and hindguts (2-3) were assayed in 1x PBS. Cell number was measured by CellTiter-Fluor^TM^ Cell Viability Assay kit (Promega_G6080). Endogenous UDP release was measured by UDP-Glo^TM^ Glycosyltransferase Assay kit (Promega_V6961). HDAC activity was detected by HDAC Cell-Based Activity Assay kit (Cayman_600150). Glucose levels were detected with the Glucose Assay Kit (Abcam_ab169559): 10 hindguts were homogenized on ice in cold Glucose Assay Buffer.

### Imaging of the digestive tract of third instar larvae

Third instar larvae were dissected in 1× PBS and fixed with 4% paraformaldehyde for 25 min at room temperature, then washed 15 min in PBT (0.1% Triton X in 1xPBS). Samples were mounted with DAPI-containing SlowFade Gold Antifade Reagent (Thermo Fisher Scientific_S36939). Fluorescence images were visualized on a Leica TSC SPE confocal microscope.

### 3D cell culture

*AKP intestinal organoids* (*VilCreER*^*T2*^ *Apc*^*fl/fl*^*, Kras*^*G12D/+*^*, Trp53*^*fl/fl*^) were received from Sansom Laboratory as a gift. Organoids were generated from age 19-week *VilCreER*^*T2*^ *Apc*^*fl/fl*^*, Kras*^*G12D/+*^*, Trp53*^*fl/fl*^ mice (Colony: RBCVAPK, ID: RCB41.1 g) and resuspended in growth factor reduced Matrigel (Corning_356231) and overlaid with Organoid Culture (OC) Media comprising: Advanced DMEM/F12 (Gibco^TM^_12634010) supplemented with 1% penicillin–streptomycin (Gibco^TM^_15070063), 10 mM HEPES (Gibco^TM^_15630056), 2 mM glutamine (Gibco^TM^_25030081), 1x N2 supplement (Gibco^TM^_17502048),1x B27 supplement (Gibco^TM^_17504044). OC Media was freshly supplemented with 100 ng/ml Noggin (PeproTech_250-38), and 50 ng/ml EGF (PeproTech_315-09).

For cell viability assessment, mechanically disrupted organoids were resuspended in Matrigel and seeded as 6 µl domes into 96-well plates in OC Media (as described above) ±drugs at 37 °C with 5% CO_2_. Control wells comprised to organoids treated with 0.1% DMSO (equivalent to final DMSO concentration in drug treated wells). Cell viability was assessed after 48 h using CellTiter-Blue® Cell Viability Assay kit (Promega_G8080). This approach can better protect research results from being influenced by observer bias. For data analysis, fluorescence intensity of drug treated wells were compared to that of control wells.

Similarly, assessment of UDP release from *AKP* organoids was performed by multiplexing cell viability assay and UDP-release assay. Briefly, mechanically disrupted organoids were seeded into 96-well plate and overlaid with OC media ± drugs as described previously. At time-point, cell viability was measured using CellTiter-Fluor™ Cell Viability Assay (Promega_G6080) and UDP-release was measured subsequently from the same plate using UDP-Glo™ Glycosyltransferase Assay (Promega_V6961). For data analysis, luminescence intensity from UDP- Glo™ assay was normalised to cell number (CellTiter-Fluor™) to compare the levels of UDP-release between different treatments. We performed all organoids quantification blinded to genotype or treatment.

### Ethical approval

Drosophila is not a protected species under UK law.

## Results

### Glucuronidation pathway is required for trametinib resistance in RAP tumours

**Fig. 1:**
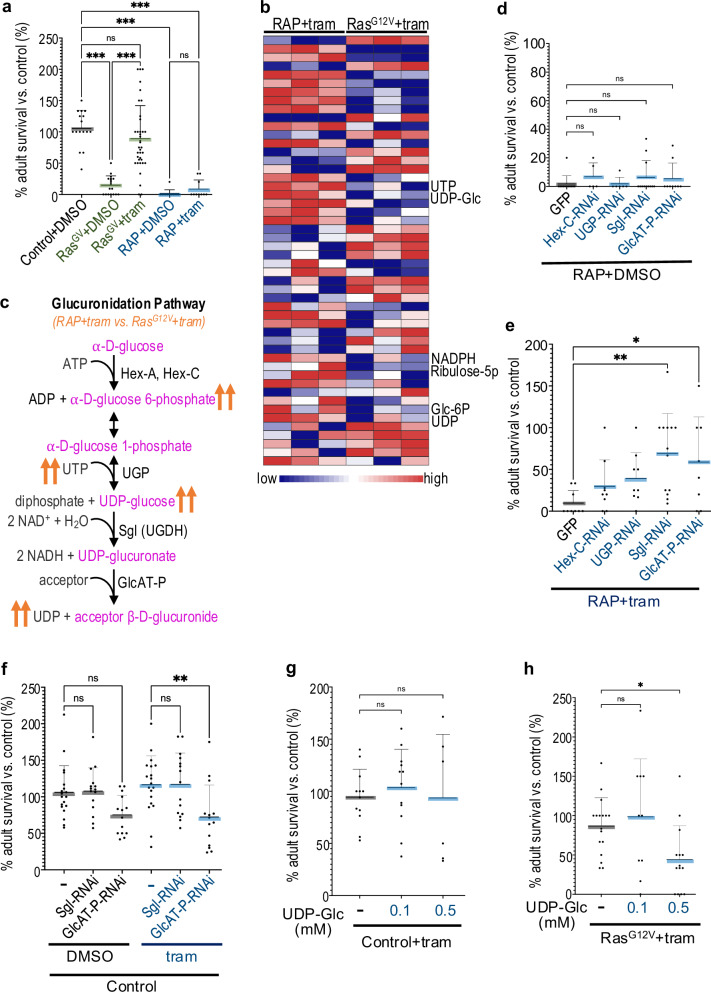
Glucuronidation pathway induces trametinib resistance in *Drosophila.* Glucuronidation was upregulated in RAP tumours compared with Ras^G12V^ tumours and led to trametinib resistance. **a**, **d**, **e**, **f**, **g**, **h** Percent survival of transgenic flies to adulthood relative to control flies was quantified in the present or absence of trametinib (1 μM) or UDP-Glc as indicated. **a** *Control* (*n* = 16), *Ras*^*G12V*^ (+DMSO *n* = 18; +tram *n* = 37) and *RAP* ( + DMSO *n* = 12; +tram *n* = 10); **d**, **e** *RAP* + *GFP* (control, +DMSO *n* = 12; +tram *n* = 9), *RAP +Hex-C-RNAi* (+DMSO *n* = 5; +tram *n* = 9), *RAP* + *UGP-RNAi* (+DMSO *n* = 6; +tram *n* = 9), *RAP +Sgl-RNAi* (+DMSO *n* = 12; +tram *n* = 13), *RAP +GlcAT-P-RNAi* (+DMSO *n* = 9; +tram *n* = 8); **f** *Control* (+DMSO *n* = 19; +tram *n* = 19), *Sgl-RNAi* (+DMSO *n* = 15; +tram *n* = 15) and *GlcAT-P-RNAi* (+DMSO *n* = 14; +tram *n* = 14); **g** *Control* (without UDP-Glc *n* = 12; +0.1 mM *n* = 12; +0.5 mM *n* = 6); **h** *Ras*^*G12V*^ (without UDP-Glc *n* = 17; +0.1 mM *n* = 10; +0.5 mM *n* = 14), error bar is a standard deviation (SD). **b** A heatmap of LC/MS showed top 50 metabolites. **c** An overview of the glucuronidation pathway. Transgene expression was induced in *Drosophila* hindguts by a *byn-GAL4* driver. Experiments were performed at 27 °C (**a**, **b**, **g**, **h**) or 29 °C (**d**, **e**, **f**). Drug concentrations indicate final food concentrations. Each data point represents a replicate. N.S *P*( > 0.12), *P(0.033), **P(0.002), ***P(0.001), and ****P( < 0.0001). *P*-values ≤ 0.033 were considered significant. All statistical data were summarized in Supplementary Table S2.

The *Drosophila* hindgut has proven a useful tool for modelling CRC including for predicting therapeutics [4]. To identify the most effective inhibitor against *Ras*^*G12V*^ tumours, we targeted transgenes to the developing hindgut using *byn-GAL4* and performed a limited FDA drug screen: the potent and specific MEK inhibitor trametinib was especially effective in reducing oncogenic *Ras*^*G12V*^-mediated transformation in the *Drosophila* hindgut, leading to increased animal survival (Supplementary Fig. S1a, b). Feeding larvae with 1 µM trametinib strongly rescued *byn>Ras*^*G12V*^-induced lethality (Fig. 1a). In contrast, a multigenic *Ras*^*G12V*^, *Apc*^*shRNA*^, *P53*^*shRNA*^ CRC model (*byn* > *RAP*)—designed to capture the three most common mutations reported for CRC—was resistant to trametinib both for animal survival (Fig. 1a) and for aspects of transformation of the hindgut proliferative zone (HPZ, see below). These data indicate an emergent resistance to trametinib in *byn* > *RAP* tumours, mirroring the trametinib resistance observed in *KRAS*-mutant CRC patients.

Recent studies have linked metabolite changes to drug resistance in liver, lung, and renal cancer models [18–20]. To identify the mechanisms of emergent trametinib resistance, we performed a metabolomics analysis by liquid chromatography–mass spectrometry (LC–MS), comparing *byn>Ras*^*G12V*^ and *byn* > *RAP* hindguts in the presence of trametinib. 143 metabolites were altered in *byn* > *RAP* tumours upon administering trametinib (Fig. 1b and Supplementary Table 1). An enrichment analysis (MetaboAnalyst 5.0, S1c) highlighted key differences between *byn* > *RAP* and *byn>Ras*^*G12V*^ in the presence of trametinib, including transfer of acetyl groups into mitochondria (TAGIM), anaerobic glycolysis (Warburg effect), glutamate metabolism, citric acid cycle, nucleotide sugar metabolism, and purine metabolism (Supplementary Fig. S1c). The strongest enrichment was for metabolites associated with the glucuronidation pathway (Fig. 1c), indicating upregulation of the pathway in *byn* > *RAP* tumours compared to *byn>Ras*^*G12V*^ tumours in the presence of trametinib. Upregulated metabolites included Glucose-6-phosphate (Glc-6P), UTP, UDP, and UDP-glucose (UDP-Glc; Fig. 1b, c and Supplementary Fig. S1d). We focussed on glucuronidation as a key correlative of resistance and a key mechanism of drug resistance: cells commonly use glucuronidation to solubilize and remove toxins including the majority of clinically relevant drugs [21]. Of note, trametinib is glucuronidated in colorectal cancer patients [22].

To investigate whether the glucuronidation pathway is essential for trametinib resistance in *byn* > *RAP* tumours, we used hindgut-targeted knockdown to reduce the activity of key glucuronidation pathway enzymes including Hexokinase C (Hex-C; human ortholog: GCK), UDP-glucose Pyrophosphorylase (UGP; UGP2), Sugarless (Sgl; UDP-glucose 6-dehydrogenase, UDGH), and Glucuronyltransferase P (GlcAT-P). The latter is a member of the human glucuronosyltransferase family that utilizes UDP-glucuronate as a substrate in *Drosophila* and has glucuronosyltransferase activity [23] similar to UDP-glucuronosyltransferases; Fig. 1c).

Knockdown of Sgl or GlcAT-P significantly rescued tumour-induced lethality in the presence of trametinib; neither knockdown impacted *byn* > *RAP* survival in the absence of trametinib or in control animals (Fig. 1d–f). Conversely, increasing the key glucuronidation pathway substrate by supplementing the food with UDP-Glc was sufficient to induce trametinib resistance in otherwise sensitive *byn>Ras*^*G12V*^ tumours (Fig. 1h). UDP-Glc did not affect survival of control animals (Fig. 1g). These data indicated that elevated glucuronidation pathway activity induces trametinib resistance in RAP tumours. Further, the data is consistent with trametinib resistance being controlled by glucuronidation.

### Glucuronidation pathway activity is enhanced by the pentose phosphate pathway

**Fig. 2:**
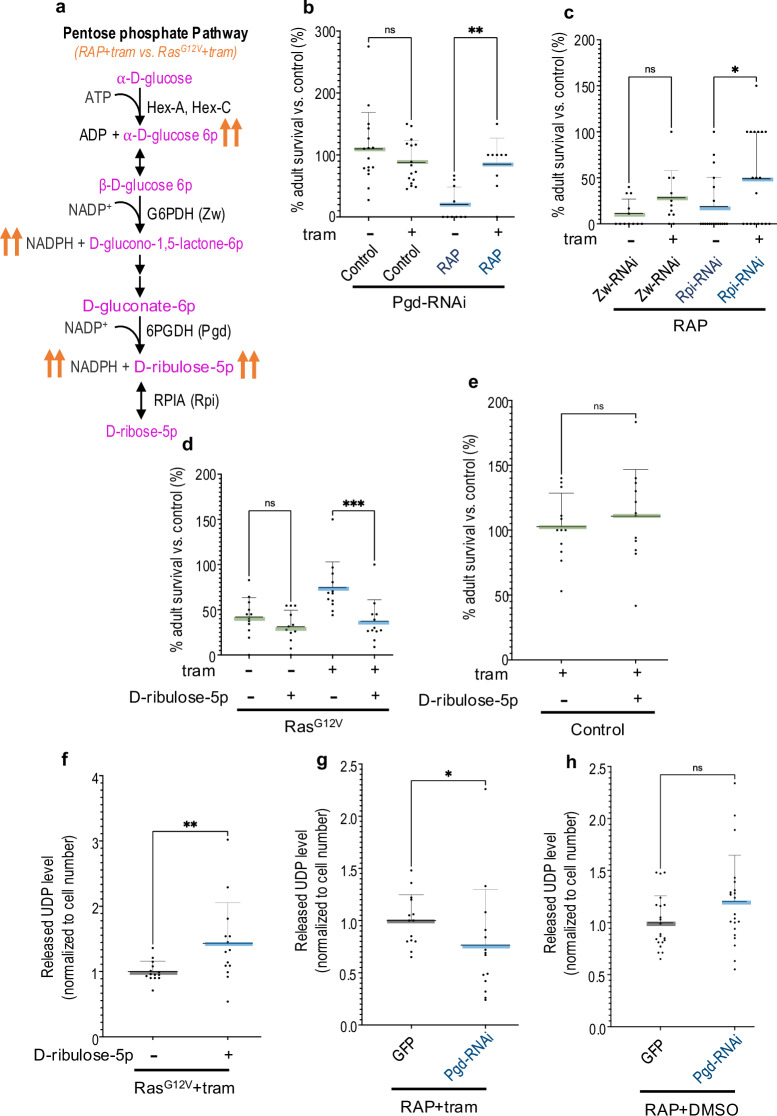
Pentose phosphate pathway enhanced trametinib resistance by promoting glucuronidation pathway. The pentose phosphate pathway was increased in RAP tumours compared with Ras^G12V^ tumours and enhanced glucuronidation of trametinib. **a** An overview of the pentose phosphate pathway. **b**–**e** Percent survival of control or tumour flies to adulthood relative to control fly was quantified in the present or absence of trametinib (1 μM), D-ribulose-5p (50 μM). **b** *Pgd-RNAi* (+DMSO *n* = 16; +tram *n* = 16) or *RAP* +*Pgd-RNAi* (+DMSO *n* = 10; +tram *n* = 9), **c** *RAP* + *Zw-RNAi* (+DMSO *n* = 12; +tram *n* = 11) or *RAP* +*Rpi-RNAi* (+DMSO *n* = 19; +tram *n* = 19), **d** *Ras*^*G12V*^ (+DMSO *n* = 12; +D-ribulose-5p *n* = 12; +tram *n* = 12; +tram/D-ribulose-5p *n* = 12), **e** *Control* (+tram *n* = 12; +tram/D-ribulose-5p *n* = 12). **f**–**h** Released UDP analysis of *Ras*^*G12V*^ (+tram *n* = 14; +tram/D-ribulose-5p *n* = 14) (**f**)*, RAP* + *GFP* (+tram *n* = 14) or *RAP* +*Pgd-RNAi* (+tram *n* = 14) (**g**), *RAP* + *GFP* ( + DMSO *n* = 21) or *RAP* +*Pgd-RNAi* (+DMSO *n* = 21) (**h**) in the present or absence of trametinib (1 μM), D-ribulose-5p (50 μM) in fly hindguts. Experiments were performed at 29 °C (**b**, **c**) or 27 °C (**d**–**h**). Error bar is a standard deviation (SD). N.S *P*( > 0.12), *P(0.033), **P(0.002), ***P(0.001), and ****P( < 0.0001). *P*-values ≤ 0.033 were considered significant. All statistical data were summarized in Supplementary Table S2.

We observed that Nicotinamide adenine dinucleotide phosphate (NADPH) and Ribulose 5-phosphate (Ribulose-5p) were also upregulated in *byn* > *RAP* tumours when compared with *byn>Ras*^*G12V*^ tumours (Fig. 1b and Supplementary Table 1). These metabolites are major components involved in the pentose phosphate pathway (PPP; Fig. 2a), another pathway involved in glucose flux. Genetic inhibition of PPP by targeted knockdown of (i) *glucose-6-phosphate dehydrogenase* (*zw*, *G6PDH*) weakly suppressed trametinib resistance in RAP tumours (Fig. 2c); (ii) *phosphogluconate dehydrogenase* (*pgd*, *6PGDH*) or *ribose-5-phosphate isomerase* (*rpi*, *RPIA*) significantly rescued RAP tumour-induced lethality in the presence of trametinib. Neither knockdown impacted survival in the absence of trametinib (Fig. 2b, c) or of control animals (Fig. 2b). Furthermore, increasing PPP substrate by feeding a high-D-ribulose-5p diet reduced trametinib sensitivity in *byn>Ras*^*G12V*^ tumours (Fig. 2d) but did not affect control animals (Fig. 2e).

The glucuronidation pathway converts circulating glucose to intracellular UDP-glucuronide (UDP-GlcA); transfer of UDP-GlcA to, e.g., a drug leads to its clearance coupled with UDP release (Fig. 1c). Hence, UDP release provides a commonly used assay for measuring levels of glucuronidation (Fig. 1c). Indeed, inhibiting glucuronidation pathway activity by knockdown of *sgl* significantly suppressed the level of released UDP in *byn* > *RAP* tumours in the present of trametinib (Supplementary Fig. S1e), supporting the use of released UDP as an indicator of glucuronidation pathway activity. High dietary D-ribulose-5p led to upregulation of released UDP levels in *byn>Ras*^*G12V*^ tumours (Fig. 2f), while knockdown of *pgd* significantly reduced UDP release in *byn* > *RAP* tumours (Fig. 2g); in the absence of trametinib, knockdown of *pgd* did not significantly reduce UDP release in *byn* > *RAP* tumours (Fig. 2h). Taken together, these results suggesting that PPP inhibits trametinib sensitivity by enhancing the activity of glucuronidation pathway.

We next explored the mechanisms by which CRC-associated gene combinations lead to glucuronidation-dependent drug resistance.

### High dietary sugar promotes glucuronidation

**Fig. 3:**
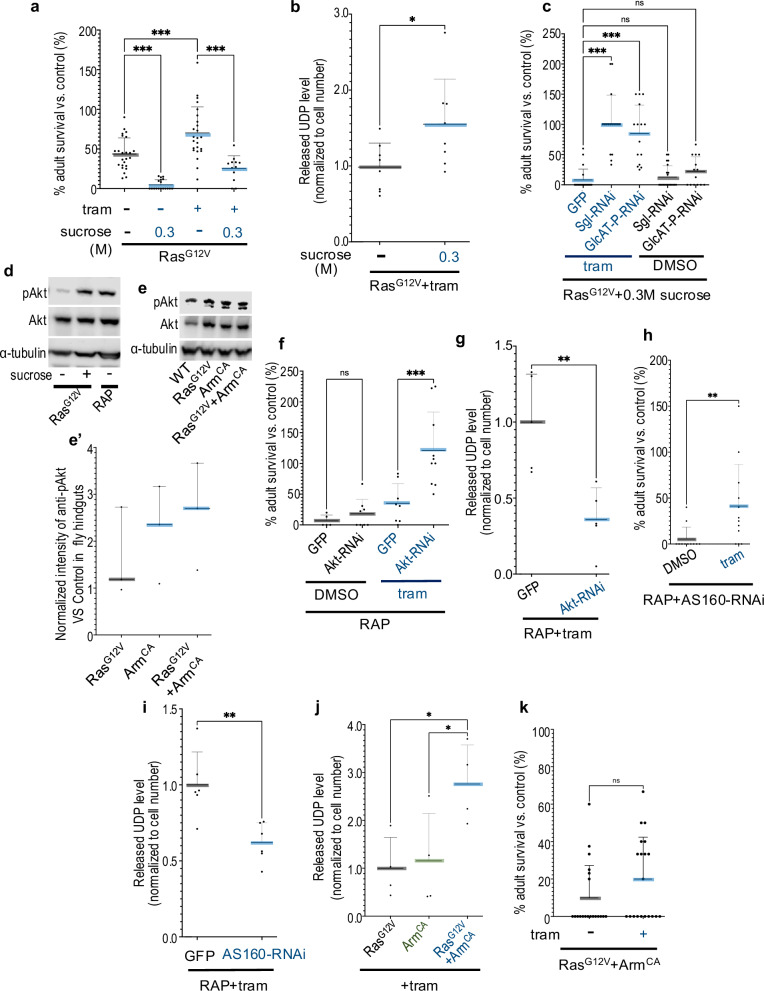
Pi3K/Akt signalling induces trametinib resistance by enhancing glucuronidation in *Drosophila.* Upregulation of Wnt signalling enhanced glucose uptake in a Pi3K/Akt-dependent manner, thereby increasing glucuronidation of trametinib. **a**, **c**, **f**, **h**, **k** Percent survival of transgenic flies to adulthood relative to control flies was quantified in the present or absence of trametinib (1 μM), sucrose, error bar is a standard deviation (SD). **a** *Ras*^*G12V*^ (+DMSO *n* = 24; +sucrose *n* = 18; +trametinib *n* = 24; +tram/sucrose *n* = 12); **c** *Ras*^*G12V*^ + *GFP* (+tram/sucrose *n* = 20), *Ras*^*G12V*^*+Sgl-RNAi* (+DMSO/sucrose *n* = 21; +tram/sucrose *n* = 17) and *Ras*^*G12V*^ *+GlcAT-P-RNAi* (+DMSO/sucrose *n* = 14; +tram/sucrose *n* = 15); **f** *RAP* + GFP ( + DMSO *n* = 5; +tram *n* = 8), *RAP* +*Akt-RNAi* (+DMSO *n* = 8; +tram *n* = 10); **h** *RAP* + *AS160-RNAi* (+DMSO *n* = 12; +tram *n* = 12); **k** *Ras*^*G12V*^ +*Arm*^*CA*^ (+DMSO *n* = 20; +tram *n* = 20) were induced in *Drosophila* hindguts. **d**, **e** Western blot analysis of *Drosophila* hindguts pAkt and Akt levels in *Ras*^*G12V*^, *RAP, Control, Arm*^*CA*^, *or Ras*^*G12V*^ +*Arm*^*CA*^ with or without sucrose. **e**’ The median normalised intensity of anti-pAKT in hindguts in each genotype compared with *Control*, error bar is a 95% confidence interval for the median, *n* = 3. **g**, **i**, **j** Released UDP analysis of *RAP* + *GFP* (+tram *n* = 5), *RAP +Akt-RNAi* (+tram *n* = 5) (**g**); *RAP* + *GFP* (+tram *n* = 6), *RAP* + *AS160-RNAi* (+tram *n* = 6) (**i**); *Ras*^*G12V*^ (+tram *n* = 4), *Arm*^*CA*^ (+tram *n* = 4) or *Ras*^*G12V*^ +*Arm*^*CA*^ (+tram *n* = 4) (**j**) with trametinib in *Drosophila* hindguts, error bar is a standard deviation (SD). Transgene expression was induced in *Drosophila* hindguts by a *byn-GAL4* driver. Increased dietary sugar led to increased glucuronidation and reduced trametinib activity, while targeting glucuronidation enzymes or Pi3K pathway activity strongly potentiated trametinib activity. Experiments were performed at 27 °C (**a**–**e**, **g** and **i**–**k**) or 29 °C (**f** and **h**). N.S *P*( > 0.12), *P(0.033), **P(0.002), ***P(0.001), and ****P( < 0.0001). *P*-values ≤ 0.033 were considered significant. All statistical data were summarized in Supplementary Table S2.

Transgenic *byn* > *RAP* hindguts displayed elevated glucose compared to *byn>Ras*^*G12V*^ hindguts (Supplementary Fig. S1f), prompting us to investigate whether elevated glucose uptake led to increased glucuronidation. Our previous work showed that high dietary sugar (HDS) promoted glucose uptake in *Ras*^*G12V*^ *csk*^*−/−*^ flies, enhancing tumour progression in eye-antennal epithelia as well as altering drug response [24–26]. Similarly, we found that HDS enhanced tumour progression in *byn>Ras*^*G12V*^ hindguts, resulting in increased animal lethality (Fig. 3a); control animals were not affected (Supplementary Fig. S2a). Importantly, HDS upregulated the level of released UDP, suggesting that HDS promoted trametinib resistance at least in part by elevating glucuronidation pathway activity (Fig. 3b).

These results raised the question as to whether HDS directs drug resistance due to its impact on glucuronidation levels *vs*. tumour progression. Inhibiting the activity of glucuronidation pathway by knockdown of key glucuronidation enzymes Sgl or GlcAT-P almost entirely suppressed the ability of HDS to reduce trametinib efficacy; knockdown of either enzyme had no effect in the absence of trametinib (Fig. 3c). Inhibiting glucuronidation pathway activity did not impact control animals including in the presence of HDS plus trametinib (Supplementary Fig. S2a). These data suggest that glucose uptake promotes trametinib resistance primarily by enhancing the glucuronidation pathway.

### PI3K/AKT signalling is required for enhancing glucuronidation pathway activity

The Pi3k/Akt signalling pathway, initiated by Ras, plays an important role in regulating glucose uptake in mammalian cells by activating AS160 [27]. Previous work demonstrated that HDS enhances glucose uptake by increasing the levels of phosphorylated Akt (pAkt) in normal Drosophila [28]. In *byn>Ras*^*G12V*^ hindgut tumours, HDS administration similarly resulted in elevated Pi3k activity, as indicated by increased pAkt levels (Fig. 3d). Interestingly, hindgut tumours in *byn* > *RAP*, even without HDS treatment, exhibited significantly higher pAkt levels compared to *byn>Ras*^*G12V*^ alone. This suggests that reduction of Apc plus P53 amplifies Ras-dependent Pi3k activity, phenocopying the effects of HDS (Fig. 3d). Further, knockdown of Akt or the AS160 ortholog Plx in *byn* > *RAP* tumours strongly reduced both glucuronidation pathway activity—based on decreased levels of released UDP—and resistance to trametinib (Figs. 1a and 3f–i).

Loss of Apc leads to cytoplasmic accumulation of ß-catenin, activating Wnt/ß-catenin signalling [29]. Overexpressing a constitutively active ß-catenin ortholog, Arm (Arm^CA^), significantly boosted both Pi3k and glucuronidation pathway activities in the presence of Ras^G12V^. This resulted in trametinib resistance in normally sensitive *byn>Ras*^*G12V*^ tumours (compare Fig. 3e, j, k with Fig. 1a). Control animals were not affected (Supplementary Fig. S2b).

These data indicate that pairing elevated Ras plus Wnt pathway activities promotes trametinib resistance by (i) promoting glucose uptake in a Pi3K/Akt dependent manner, which in turn (ii) enhances glucuronidation pathway activity and (iii) resistance to trametinib. Consistent with this view, pharmacological inhibition of Pi3K/Akt with the compound LY294002 significantly increased trametinib sensitivity in *byn* > *RAP* animals (Supplementary Fig. S2c) at doses that did not impact control animals (Supplementary Fig. S2d).

### Glucuronidation promoted trametinib resistance in mouse AKP organoids

**Fig. 4:**
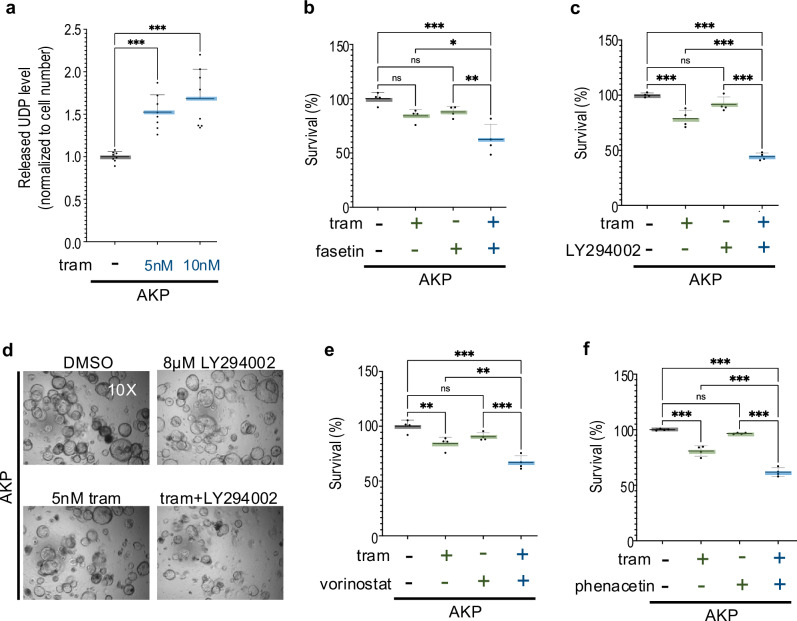
Deacetylation, glucuronidation lead to trametinib resistance in mouse AKP organoids. Glucuronidation contributed to trametinib resistance in mouse AKP organoids. **a** Released UDP analysis of mouse AKP organoids in the present of trametinib. **b**, **c**, **e**, **f** Percent survival of AKP organoids relative to control was quantified in the present or absence of trametinib (5 nM), fasentin (30 μM), LY294002 (8 μM), vorinostat (0.5 μM) or phenacetin (100 μM). Error bar is a standard deviation (SD), *n* = 4. Data points display technical replicates. **d** Representative images showing the impact of drugs on AKP organoids, magnification 10X. Targeting glucuronidation led to increased effectiveness of trametinib. N.S *P*( > 0.12), *P(0.033), **P(0.002), ***P(0.001), and ****P( < 0.0001). *P*-values ≤ 0.033 were considered significant. All statistical data were summarized in Supplementary Table S2.

To assess if our Drosophila data is relevant to drug response in a mammalian CRC platform, we investigated whether glucuronidation promotes trametinib resistance in a mouse *VilCreER*^*T2*^, *Apc*^*fl/fl*^, *Kras*^*G12D/+*^, *Trp53*^*fl/fl*^ transgenic cell line derived from the small intestine. This “*AKP*” tumour organoid exhibited a high level of released UDP in the present of trametinib (Fig. 4a). Consistent with our *Drosophila* results, adding the key glucuronidation pathway substrate UDP-Glc to the media inhibited response to high-dose trametinib (20 nM) in AKP tumour organoids (Supplementary Fig. S3a).

*AKP* organoids proved moderately sensitive to trametinib (Fig. 4b–f). Suppressing glucose uptake with (i) the GLUT1/GLUT4 inhibitor fasentin or (ii) the Pi3k/Akt inhibitors LY294002 and alpelisib significantly increased trametinib sensitivity. Single agents had no effect on tumour organoid expansion (Fig. 4b–d, Supplementary Fig. S3b–g). These data suggest that elevated glucuronidation pathway activity can suppress *AKP* organoids’ response to trametinib.

### Glucuronidation was blocked by targeting deacetylation or reducing blood glucose

Interfering with regulatory steps in the glucuronidation pathway including Pi3K signalling and glucuronidation enzymes elevated trametinib activity in our CRC models. However, combining inhibition of these pathways with inhibition of MEK can lead to unwanted and significant toxicity [21, 30–34]. In cancer patients, glucuronidation of trametinib occurs in a two-step process: deacetylation followed by glucuronidation of two distinct moieties [22]. Histone deacetylases (HDACs) deacetylate both histone proteins and non-histone cellular substrates that govern a wide array of disease processes including tumour progression and tumour therapy, and HDAC inhibitors are a staple of cancer treatment [35, 36]. We therefore used both genetics and drugs to assess whether blocking deacetylation could provide a clinically accessible target to reducing trametinib glucuronidation.

**Fig. 5:**
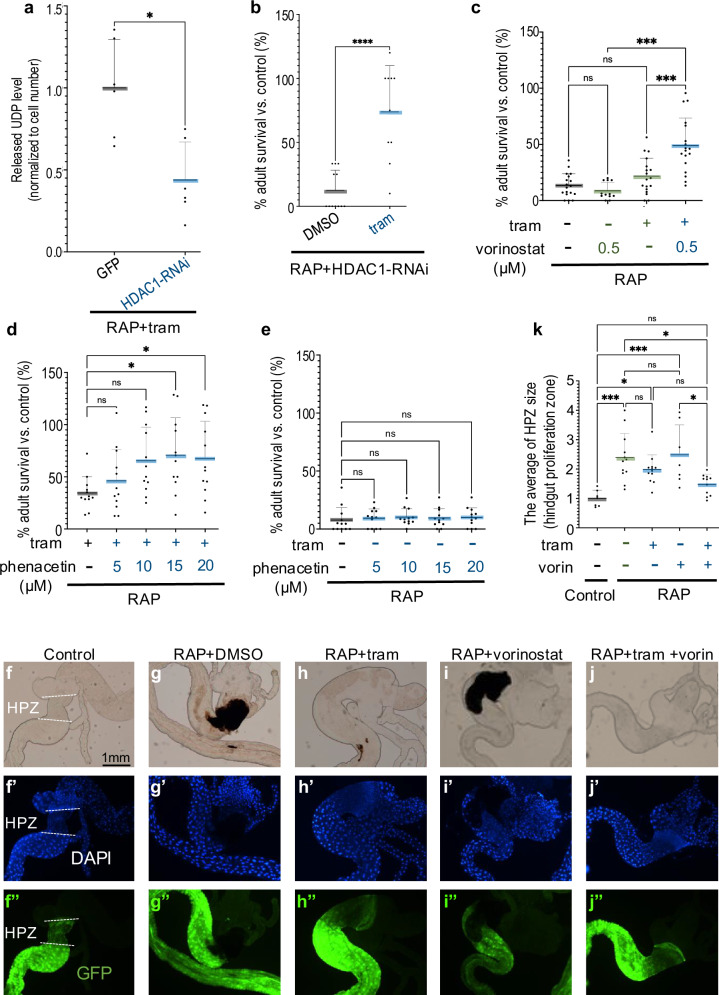
HDAC1 is required for glucuronidation of trametinib in *Drosophila.* Trametinib was deacetylated by HDAC1 in *Drosophila*. **a** Released UDP analysis of *RAP* + *GFP* (+tram *n* = 6) or *RAP* + *HDAC1-RNAi* (+tram *n* = 6) with trametinib in *Drosophila* hindguts. **b**–**e** Percent survival of adult tumour flies relative to control flies was quantified in the present or absence of trametinib (1 μM), vorinostat (0.5 μM) or phenacetin. **b** *RAP* + *HDAC1-RNAi* (+DMSO *n* = 12; +tram *n* = 10), **c**–**e** *RAP* ( + DMSO *n* = 18; +vorinostat *n* = 12; +tram *n* = 18; +tram/vorinostat *n* = 18) (**c**); *RAP* (+tram *n* = 12; +tram/5 μM phenacetin *n* = 12; +tram/10 μM phenacetin *n* = 12; +tram/15 μM phenacetin *n* = 12; +tram/20 μM phenacetin *n* = 12) (**d**); *RAP* (+DMSO *n* = 12; +5 μM phenacetin *n* = 12; +10 μM phenacetin *n* = 12; +15 μM phenacetin *n* = 12; +20 μM phenacetin *n* = 12) (**e**). Reduced HDAC activity led to reduced trametinib-dependent UDP release. **f**–**j** Images of the digestive tract of third instar larvae in the present or absence of trametinib (1 μM), vorinostat (0.5 μM) which include the hindgut proliferation zone (HPZ). Nuclei are visualized with 4′,6-diamidino-2-phenylindole (DAPI) staining, hindgut is marked by GFP. Scale bar 1 mm. **k** The average of hindgut proliferation zone (HPZ) size was measured by Fiji ImageJ and quantified as relative size to control hindgut. *Control* (*n* = 7), *RAP* (+DMSO *n* = 12; +vorinostat *n* = 7; +tram *n* = 12; +tram/vorinostat *n* = 11). Experiments were performed at 27 °C (**a**, **c** and **d**–**k**) or 29 °C (**b**). Error bar is a standard deviation (SD). N.S *P*( > 0.12), *P(0.033), **P(0.002), ***P(0.001), and ****P( < 0.0001). *P*-values ≤ 0.033 were considered significant. All statistical data were summarized in Supplementary Table S2. Transgene expression was induced in *Drosophila* hindguts by a *byn-GAL4* driver. Reducing deacetylation/glucuronidation with vorinostat increased trametinib’s ability to rescue hindgut size.

Knockdown of the *Drosophila* deacetylase HDAC1 significantly suppressed trametinib glucuronidation in *byn* > *RAP* animals as determined by reduced UDP release (Fig. 5a). The result was significantly increased sensitivity to trametinib and improved rescue of *byn* > *RAP* survival (Fig. 5b). Similarly, co-feeding *byn* > *RAP* animals with the drug vorinostat (SAHA)—a clinically relevant HDAC inhibitor that binds to the active site of histone deacetylases [37]—significantly reduced trametinib resistance. Vorinostat had no detectable effect as a single agent (Fig. 5c). This suggests that deacetylation is indeed required for glucuronidation and for trametinib resistance in *byn* > *RAP* flies. We also examined histone acetyltransferases (HATs), which suppress the HDAC enzymes responsible for adding acetyl groups. Knockdown of *Drosophila* P300 histone acetyltransferase *nejire* (*nej*) significantly suppressed trametinib sensitivity in *byn>Ras*^*G12V*^ tumours, while it did not affect tumour induced lethality (Supplementary Fig. S4b).

Together, this data supports a model in which deacetylation is indeed required for glucuronidation of trametinib, providing an accessible point of interference. Of note, unlike glucuronidation, the baseline activity of HDAC did not significantly differ between *byn>Ras*^*G12V*^ and *byn* > *RAP* hindguts as assessed by a cell permeable, fluorescent HDAC substrate (Supplementary Fig. S4a). This suggests that, while deacetylation is a necessary first step for glucuronidation of trametinib, it likely does not account for the differential drug sensitivity observed between *byn>Ras*^*G12V*^ and *byn* > *RAP* animals.

**Fig. 6:**
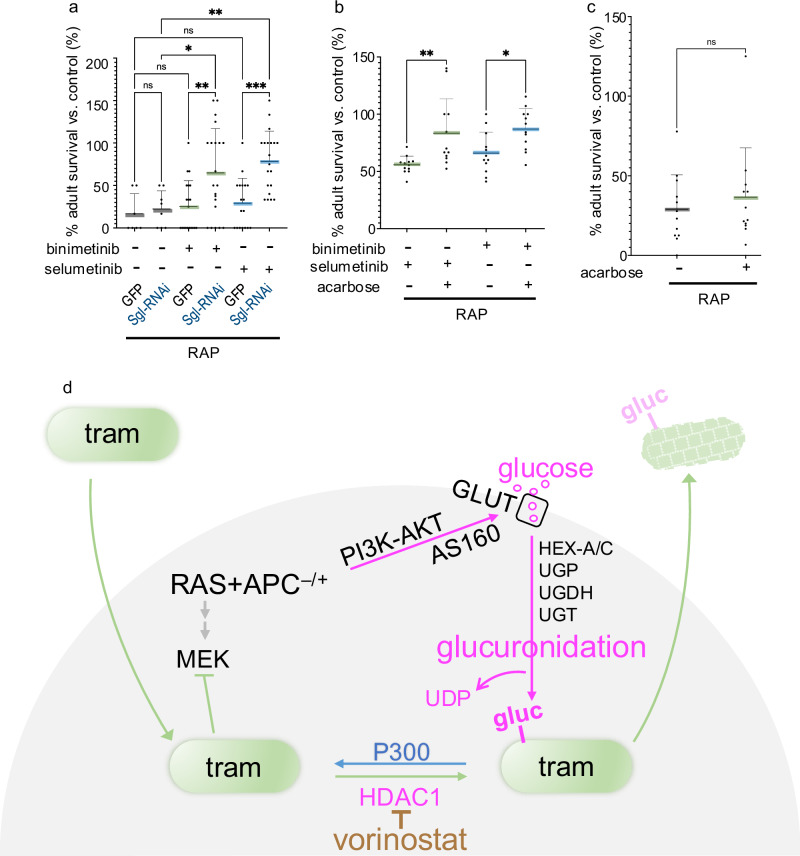
Reducing blood glucose levels suppressed drug resistance in RAP tumours. Reducing blood glucose levels by feeding acarbose suppressed the glucuronidation of trametinib. **a**–**c** Percent survival of control or tumour flies to adulthood relative to control fly was quantified in the present or absence of binimetinib (8 μM), selumetinib (8 μM), or acarbose (15 μM). **a** *RAP* ( + DMSO *n* = 7; +binimetinib *n* = 20; +selumetinib *n* = 18) or *RAP* +*Sgl-RNAi* (+DMSO *n* = 8; +binimetinib *n* = 21; +selumetinib *n* = 20), **b** *RAP* (+selumetinib *n* = 12; +selumetinib/acarbose *n* = 12; +binimetinib *n* = 12; +binimetinib/acarbose). **c** *RAP* ( + DMSO *n* = 12; +acarbose *n* = 12). **a**–**c** Experiments were performed at 27 °C. Error bar is a standard deviation (SD). N.S *P*( > 0.12), *P(0.033), **P(0.002), ***P(0.001), and ****P( < 0.0001). *P*-values ≤ 0.033 were considered significant. All statistical data were summarized in Supplementary Table S2. **d** Schematic summary. Trametinib (tram) is a potent MEK inhibitor with the demonstrated preclinical ability to block RAS pathway signalling and oncogenic transformation. Pairing activated RAS and WNT activities leads to activation of PI3K/AKT signalling, AS160, and GLUT1/4 to increase glucose flux into cells. The result is elevated glucuronidation and elimination of trametinib. Potential therapeutic targets include HDAC1: deacetylation is an obligatory pre-step required for glucuronidation of some drugs including trametinib. This reaction can be reversed by P300.

We also examined the impact of a competitor drug. Similar to trametinib, the acetamide-based drug phenacetin is modified by deacetylation and glucuronidation [38]. Administered as a single agent, phenacetin had no effect on *byn* > *RAP* survival (Fig. 5e). However, combining trametinib with phenacetin alleviated drug resistance to rescue animals in a dose-dependent manner (Fig. 5d). This data further supports the view that, similar to human patients [22], *byn* > *RAP* animals require a two-step modification to suppress trametinib: deacetylation followed by glucuronidation. Also, inhibiting the key glucuronidation pathway enzyme Sgl by targeted knockdown significantly increased *byn* > RAP sensitivity to MEK inhibitors binimetinib and selumetinib (Fig. 6a). Unlike trametinib, binimetinib and selumetinib undergo direct glucuronidation without an initial deacetylation step, and vorinostat was ineffective when paired with either drug.

To further target precursors required for glucuronidation we administered acarbose, an oral alpha-glucosidase inhibitor used in managing type 2 diabetes mellitus and previously shown to reduce glucose in Drosophila [39]. In *byn* > *RAP* tumours, acarbose significantly enhanced sensitivity to multiple drugs (Fig. 6b); feeding acarbose alone did not impact tumour growth (Fig. 6c). These data suggest that suppression of blood glucose levels provides an additional practical and potent strategy to counteract drug metabolism within tumour cells.

The impact of glucuronidation on drug response extended beyond animal survival. Targeting the hindgut proliferative zone (HPZ) in *byn* > *RAP* animals led to significant overgrowth compared to control animals (Fig. 5g compared to 5 f, quantified in 5k). Consistent with our adult survival assay, inhibiting deacetylation (vorinostat) in the presence of trametinib significantly suppressed overgrowth of the HPZ in *byn* > *RAP* tumours; trametinib or vorinostat alone did not have a strong effect (Fig. 5h–j compared to 5 g, quantified in 5k). Together, our data indicate that the activity of the glucuronidation pathway is enhanced by reducing Apc plus P53 activities in genotypically *byn* > *RAS*^*G12V*^ hindguts, leading to emergent drug resistance.

### Altering glucuronidation impacted drug response in mouse organoids

Our findings extended to *Apc*^*fl/fl*^, *Kras*^*G12D/+*^, *Trp53*^*fl/fl*^ (AKP) mouse organoids. Similar to our Drosophila models, inhibiting trametinib deacetylation by HDAC inhibitor (vorinostat) or via a competing substrate (phenacetin) also significantly suppressed trametinib resistance in mouse AKP tumour organoids; again, single agents had no effect in the absence of trametinib (Fig. 4e, f, Supplementary Fig. S3e, h). These data indicate that, similar to fly RAP, deacetylation and glucuronidation are required for trametinib resistance in mouse AKP tumour organoids. HDAC inhibitors are well tolerated in the clinics, and this data provides a clinically accessible route to blocking glucuronidation of drugs such as trametinib that require a two-step modification.

## Discussion

Drug resistance in KRAS-dependent CRC patients remains one of the cancer field’s most persistent challenges. In this study, we demonstrate a mechanism by which CRC tumours achieve resistance to targeted therapies by elevating glucuronidation-mediated drug metabolism. We focused on trametinib, a potent MEK inhibitor that consistently failed to show significant clinical efficacy in KRAS-mutant CRC patients. We confirmed previous observations [22] that trametinib is first deacetylated to prepare the drug for glucuronidation, which in turn resulted in inactivation/elimination of trametinib in a *byn* > *RAP* hindgut tumour model. This upregulation of glucuronidation pathway activity was achieved by elevated Ras plus Wnt pathway activities in *RAP* tumours, which in turn increased glucose uptake in a Pi3K/Akt-dependent manner.

Blocking the Ras-Wnt-Pi3K-deacetylation/glucuronidation network at any one of several points along the network strongly suppressed drug resistance in *byn* > *RAP* tumours (Fig. 6d). For example, we provide evidence that trametinib is deacetylated by Histone Deacetylase 1 (HDAC1); combining trametinib with the HDAC1 inhibitor vorinostat proved potent in both Drosophila and mouse RAS-APC-P53 CRC models, addressing the most frequent three-mutation combination reported for CRC patients. Expanding this network, we demonstrate that—compared with *byn>Ras*^*G12V*^ tumours—pentose phosphate pathway (PPP) is elevated in *byn* > *RAP* tumours, leading to reduced trametinib sensitivity by enhancing the activity of glucuronidation pathway. Therefore, it will be interesting to explore the molecular link between PPP and glucuronidation in future studies. Our findings indicate that glucuronidation—a major drug detoxification pathway—is upregulated in the context of oncogenic transformation and that this regulation is reversible by intervening at several points along the network. Future work will need to determine whether similar mechanisms are exploitable in patients, who have additional mutations that can alter tumour networks as well as drug targeting.

More than 70 therapeutic agents have been reported as metabolised by glucuronidation. Glucuronidation has been considered as a potential target of anticancer drug resistance including for colon cancer [21, 40], but mechanisms for regulating the pathway have been unclear and the large number of UDP-glucuronosyltransferases (UGTs) has made them poor candidates for targeting the pathway. Our study demonstrates that elevated Ras/Erk plus Wnt/ß-catenin signalling upregulates Pi3K/Akt/Glut1 activity: the result is increased glucose uptake, enhanced glucuronidation, and emergent drug resistance in *byn>Ras*^*G12V*^ tumours (Fig. 6d). Therapeutic targets include members of the WNT/β-catenin and PI3K/AKT pathways; for drugs such as trametinib that require an initial deacetylation step, we demonstrate the utility of HDAC inhibitors such as vorinostat as adjunct therapeutics in preclinical models. We hypothesise that this leads to a functional reduction of RAS pathway signalling and tumour progression.

This two-step glucuronidation process also suggests a mechanism by which trametinib remains stable-but-inactive in the body: initial rapid deacetylation of trametinib keeps a metabolite in circulation until a slower glucuronidation step leads to its clearance. This view is consistent with previously described distribution of trametinib metabolites in patients [22],and could explain trametinib’s persistence in the body despite poor effectiveness.

Increased glucose uptake is a characteristic of cancer cells, and aerobic glycolysis efficiently produces ATP synthesis that promotes cell proliferation, known as the Warburg effect [41]. Glycolysis also influences drug response including chemotherapeutics, immune checkpoint inhibitors and small molecule therapeutics through induction of autophagy, epithelial-mesenchymal transition (EMT), and by enhancing glycolytic enzymes impact on nonenzymatic activities [41, 42]. Our data show that the high levels of glucose in transformed cells can also activate glucuronidation pathway activity, enhancing drug metabolism in canonical *RAS-APC-P53* CRC tumours. Of note, a high sugar diet was sufficient to activate glucuronidation in *Ras*^*G12V*^ tumours, suggesting that high sugar diets can directly impact a patient’s response to anticancer drugs. We found that reducing blood glucose levels by the administration of type 2 diabetes mellitus drug acarbose significantly enhanced sensitivity to MEK inhibitors trametinib, binimetinib and selumetinib in *RAP* tumours, providing a practical strategy to counteract drug metabolism within tumour cells that elevate RAS/WNT.

In summary, our study suggests multiple points to target along the emergent RAS-WNT-glucuronidation network for re-sensitizing tumours to targeted therapies, providing insight into the long-observed difference between genetic and chemical deletion of a therapeutic target as well as preclinical and clinical models that differ in genomic complexity.

## Supplementary information

Supplemental Figure 1

Supplemental Figure 2

Supplemental Figure 3

Supplemental Figure 4

Supplemental Table S1

Supplemental Table S2

Supplemental Table S3

## Data Availability

All raw data are available from the corresponding author upon request.
