# Supplementary material for: Colon cancer cells evade drug action by enhancing drug metabolism
Source: Oncogene. 2025 Jul 10;44(36):3284–96. doi: 10.1038/s41388-025-03472-3 (PMC12399418; doi:10.1038/s41388-025-03472-3)

a

Compounds	Targets	Rescue rate (Compound/DMSO)
Trametinib	Mek	52.67%
Selumetinib	Mek	23.73%
Binimetinib	Mek	27.31%
Dabrafenib	Raf	-
Dasatinib	Raf/Multikinase	-
Vemurafenib	Raf	-
Sorafenib	Ras/Multikinase	-

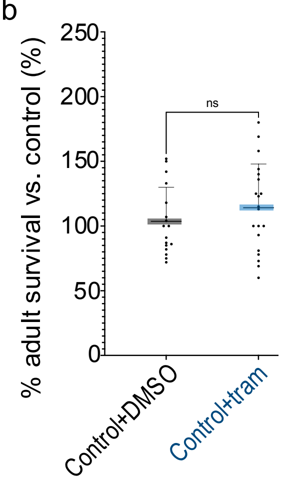

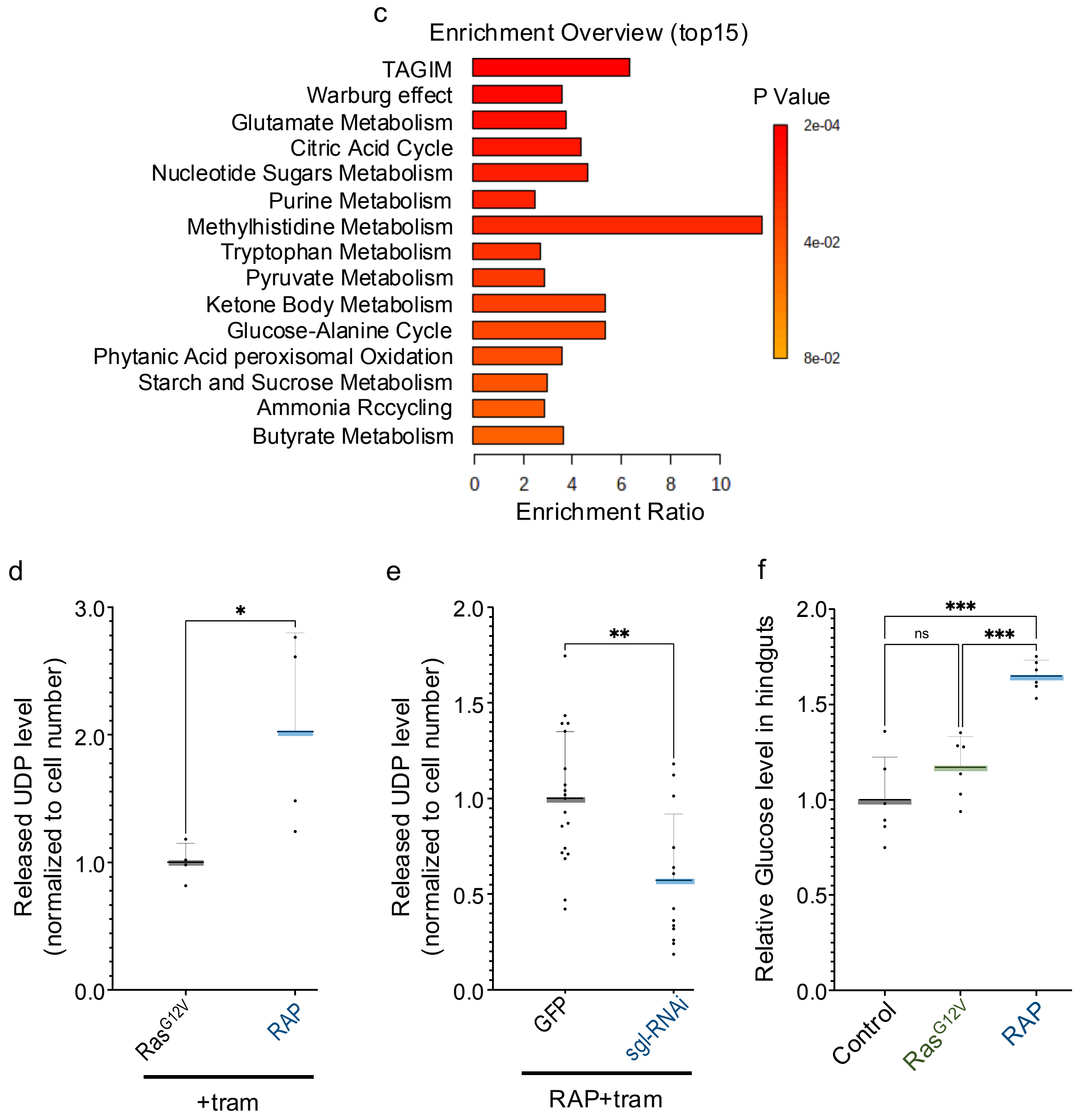

Supplement: Supplementary file 1 — Supplemental Figure 1 [file 41388_2025_3472_MOESM1_ESM.pdf]
