# Supplementary figures and images for: Colon cancer cells evade drug action by enhancing drug metabolism
Source: Oncogene. 2025 Jul 10;44(36):3284–96. doi: 10.1038/s41388-025-03472-3 (PMC12399418; doi:10.1038/s41388-025-03472-3)

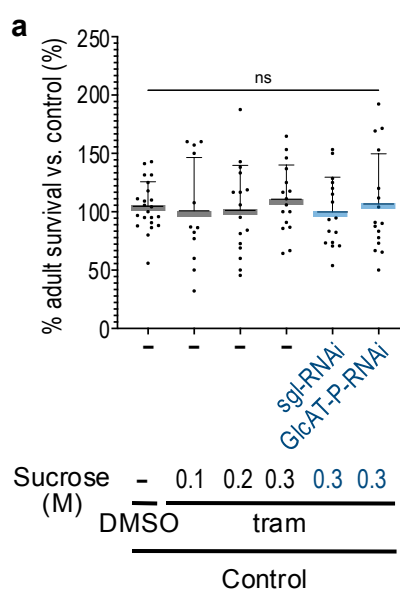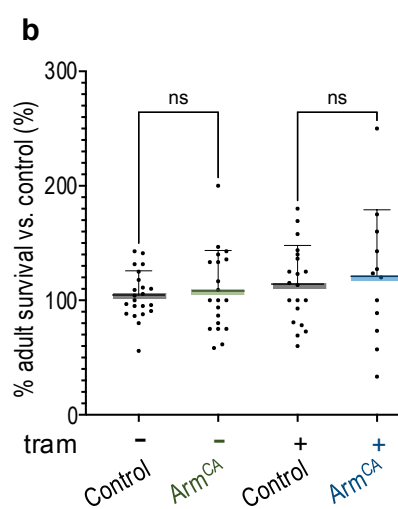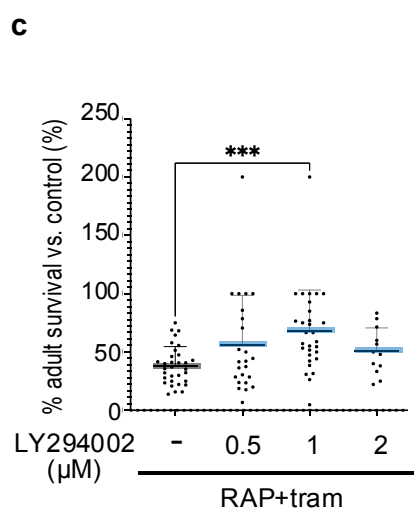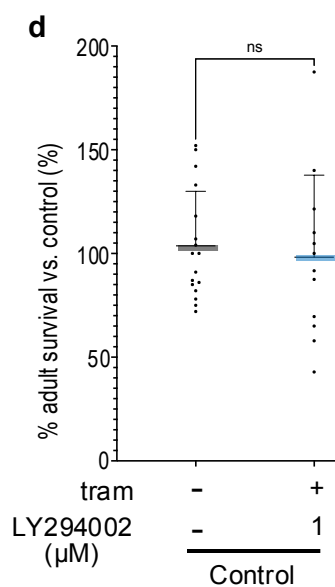

Supplement: Supplementary file 2 — Supplemental Figure 2 [file 41388_2025_3472_MOESM2_ESM.pdf]

**a**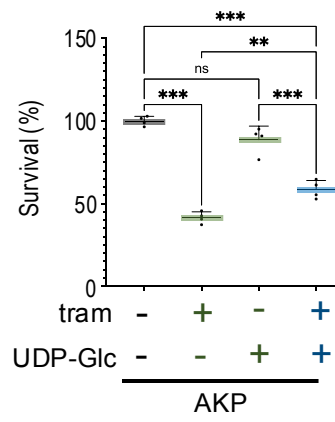**b**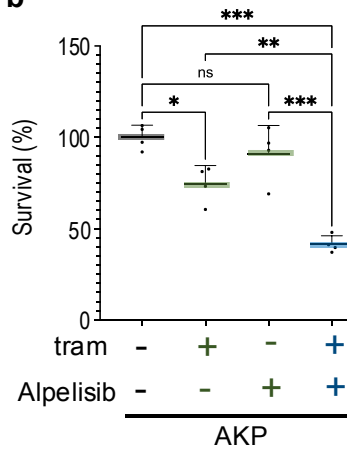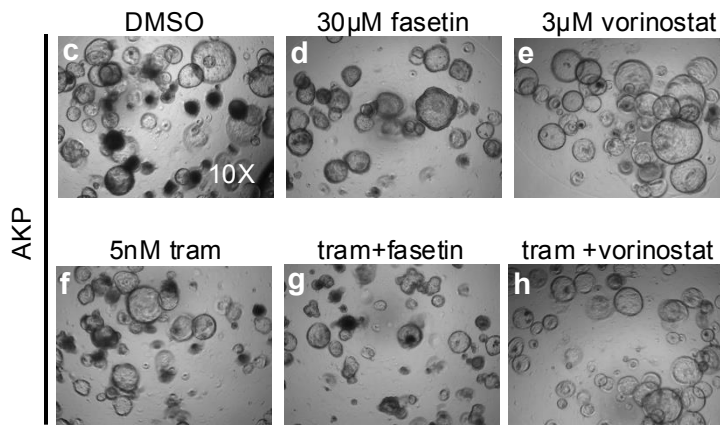

Supplement: Supplementary file 3 — Supplemental Figure 3 [file 41388_2025_3472_MOESM3_ESM.pdf]

**a**

Relative HDAC activity in hindguts

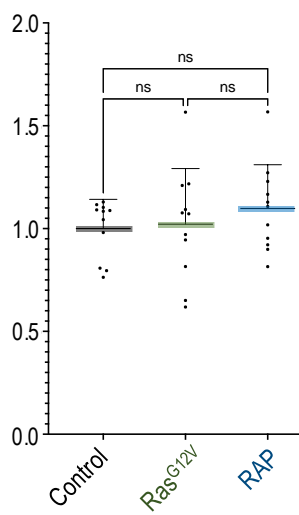

**b**

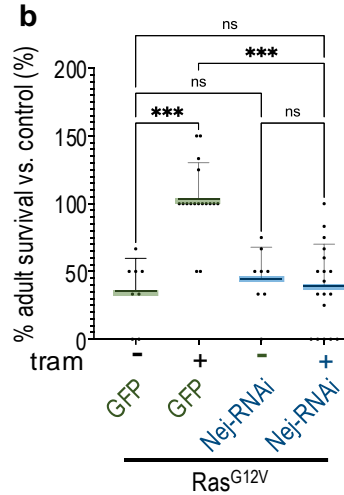

Supplement: Supplementary file 4 — Supplemental Figure 4 [file 41388_2025_3472_MOESM4_ESM.pdf]
