# Supplementary material for: Colon cancer cells evade drug action by enhancing drug metabolism
Source: Oncogene. 2025 Jul 10;44(36):3284–96. doi: 10.1038/s41388-025-03472-3 (PMC12399418; doi:10.1038/s41388-025-03472-3)
Supplement: Supplementary file 7 — Supplemental Table S3 [file 41388_2025_3472_MOESM7_ESM.pdf]

Colon Cancer Cells Evade Drug Activity by Enhancing Drug Metabolism
Detailed genotypes:

Figure 1:
+/+ or Y; +/+; byn-Gal4, UAS-GFP, tub-Gal80^{TS}*/+* (a, f and g), *+/+ or Y; UAS-Ras*^{G12V}*/+; byn-Gal4, UAS-GFP, tub-Gal80*^{TS}*/+* (a, b and h), *+/+ or Y; UAS-Ras*^{G12V}*, Apc-RNAi, UAS-P53-RNAi/+; byn-Gal4, UAS-GFP, tub-Gal80*^{TS}*/+* (a and b), *+/+ or Y; UAS-Ras*^{G12V}*, Apc-RNAi, UAS-P53-RNAi/UAS-GFP; byn-Gal4, UAS-GFP, tub-Gal80*^{TS}*/+* (b, d and e), *+/+ or Y; UAS-Ras*^{G12V}*, Apc-RNAi, UAS-P53-RNAi/UAS-Hex-C-RNAi; byn-Gal4, UAS-GFP, tub-Gal80*^{TS}*/+* (d and e), *+/+ or Y; UAS-Ras*^{G12V}*, Apc-RNAi, UAS-P53-RNAi/UAS-UGP-RNAi; byn-Gal4, UAS-GFP, tub-Gal80*^{TS}*/+* (d and e), *+/+ or Y; UAS-Ras*^{G12V}*, Apc-RNAi, UAS-P53-RNAi/UAS-Sgl-RNAi; byn-Gal4, UAS-GFP, tub-Gal80*^{TS}*/+* (d and e), *+/+ or Y; UAS-Ras*^{G12V}*, Apc-RNAi, UAS-P53-RNAi/UAS-GlcAT-P-RNAi; byn-Gal4, UAS-GFP, tub-Gal80*^{TS}*/+* (d and e), *+/+ or Y; +/UAS-Sgl-RNAi; byn-Gal4, UAS-GFP, tub-Gal80*^{TS}*/+* (f), *+/+ or Y; +/UAS-GlcAT-P-RNAi; byn-Gal4, UAS-GFP, tub-Gal80*^{TS}*/+* (f).

Figure 2:
+/+ or Y; UAS-pgd-RNAi/+; byn-Gal4, UAS-GFP, tub-Gal80^{TS}*/+* (b), *+/+ or Y; UAS-Ras*^{G12V}*, Apc-RNAi, UAS-P53-RNAi/UAS-pgd-RNAi; byn-Gal4, UAS-GFP, tub-Gal80*^{TS}*/+* (b g and h), *+/+ or Y; UAS-Ras*^{G12V}*, Apc-RNAi, UAS-P53-RNAi/+; byn-Gal4, UAS-GFP, tub-Gal80*^{TS}*/ UAS-zw-RNAi* (c), *+/+ or Y; UAS-Ras*^{G12V}*, Apc-RNAi, UAS-P53-RNAi/UAS-rpi-RNAi; byn-Gal4, UAS-GFP, tub-Gal80*^{TS}*/+* (c), *+/+ or Y; UAS-Ras*^{G12V}*/+; byn-Gal4, UAS-GFP, tub-Gal80*^{TS}*/+* (d and f), *+/+ or Y; +/+; byn-Gal4, UAS-GFP, tub-Gal80*^{TS}*/+* (e), *+/+ or Y; UAS-Ras*^{G12V}*, Apc-RNAi, UAS-P53-RNAi/UAS-GFP; byn-Gal4, UAS-GFP, tub-Gal80*^{TS}*/+* (g and h).

Figure 3:
+/+ or Y; UAS-Ras^{G12V}*/+; byn-Gal4, UAS-GFP, tub-Gal80*^{TS}*/+* (a, b and d), *+/+ or Y; UAS-Ras*^{G12V}*/UAS-GFP; byn-Gal4, UAS-GFP, tub-Gal80*^{TS}*/+* (c, e, e', j and k), *+/+ or Y; UAS-Ras*^{G12V}*/UAS-Sgl-RNAi; byn-Gal4, UAS-GFP, tub-Gal80*^{TS}*/+* (c), *+/+ or Y; UAS-Ras*^{G12V}*/UAS-GlcAT-P-RNAi; byn-Gal4, UAS-GFP, tub-Gal80*^{TS}*/+* (c), *+/+ or Y; +/UAS-GFP; byn-Gal4, UAS-GFP, tub-Gal80*^{TS}*/+* (c, e and e'), *+/+ or Y; UAS-Ras*^{G12V}*, Apc-RNAi, UAS-P53-RNAi/+; byn-Gal4, UAS-GFP, tub-Gal80*^{TS}*/+* (d), *+/+ or Y; UAS-Ras*^{G12V}*, Apc-RNAi, UAS-P53-RNAi/UAS-GFP; byn-Gal4, UAS-GFP, tub-Gal80*^{TS}*/+* (f, g and i), *+/+ or Y; UAS-Ras*^{G12V}*, Apc-RNAi, UAS-P53-RNAi/UAS-Akt-RNAi; byn-Gal4, UAS-GFP, tub-Gal80*^{TS}*/+* (f and g), *+/+ or Y; UAS-Ras*^{G12V}*, Apc-RNAi, UAS-P53-RNAi/UAS-AS160-RNAi; byn-Gal4, UAS-GFP, tub-Gal80*^{TS}*/+* (h and i), *UAS-Arm*^{CA}*/+ or Y; UAS-GFP/+; byn-Gal4, UAS-GFP, tub-Gal80*^{TS}*/+* (e, e', j and k), *UAS-Arm*^{CA}*/+ or Y; UAS-Ras*^{G12V}*/+; byn-Gal4, UAS-GFP, tub-Gal80*^{TS}*/+* (e, e', j and k).

Figure 4:
VilCreER^{T2} *Apc*$^{fl/fl}$, *Kras*$^{G12D/+}$, *Trp53*$^{fl/fl}$ (A-F).

Figure 5:
+/+ or Y; UAS-Ras^{G12V}*, Apc-RNAi, UAS-P53-RNAi/UAS-GFP; byn-Gal4, UAS-GFP, tub-Gal80*^{TS}*/+* (a), *+/+ or Y; UAS-Ras*^{G12V}*, Apc-RNAi, UAS-P53-RNAi/UAS-HDAC1-RNAi; byn-*

Gal4, UAS-GFP, tub-Gal80^{TS}*/+* (a and b), *+/+ or Y; UAS-Ras*^{G12V}*, Apc-RNAi, UAS-P53-RNAi/+; byn-Gal4, UAS-GFP, tub-Gal80*^{TS}*/+* (c, d, e and g-k), *+/+ or Y; +/+; byn-Gal4, UAS-GFP, tub-Gal80*^{TS}*/+* (f and k).

Figure 6:
+/+ or Y; UAS-Ras^{G12V}*, Apc-RNAi, UAS-P53-RNAi/UAS-GFP; byn-Gal4, UAS-GFP, tub-Gal80*^{TS}*/+* (a), *+/+ or Y; UAS-Ras*^{G12V}*, Apc-RNAi, UAS-P53-RNAi/UAS-Sgl-RNAi; byn-Gal4, UAS-GFP, tub-Gal80*^{TS}*/+* (a), *+/+ or Y; UAS-Ras*^{G12V}*, Apc-RNAi, UAS-P53-RNAi/+; byn-Gal4, UAS-GFP, tub-Gal80*^{TS}*/+* (b and c).

Supplementary Figure 1:
+/+ or Y; UAS-Ras^{G12V}*/+; byn-Gal4, UAS-GFP, tub-Gal80*^{TS}*/+* (a, c, d and f), *+/+ or Y; +/+; byn-Gal4, UAS-GFP, tub-Gal80*^{TS}*/+* (b and f), *+/+ or Y; UAS-Ras*^{G12V}*, Apc-RNAi, UAS-P53-RNAi/+; byn-Gal4, UAS-GFP, tub-Gal80*^{TS}*/+* (c, d and f), *+/+ or Y; UAS-Ras*^{G12V}*, Apc-RNAi, UAS-P53-RNAi/UAS-GFP; byn-Gal4, UAS-GFP, tub-Gal80*^{TS}*/+* (e), *+/+ or Y; UAS-Ras*^{G12V}*, Apc-RNAi, UAS-P53-RNAi/UAS-Sgl-RNAi; byn-Gal4, UAS-GFP, tub-Gal80*^{TS}*/+* (e).

Supplementary Figure 2:
+/+ or Y; +/+; byn-Gal4, UAS-GFP, tub-Gal80^{TS}*/+* (a, b and d), *+/+ or Y; +/UAS-Sgl-RNAi; byn-Gal4, UAS-GFP, tub-Gal80*^{TS}*/+* (a), *+/+ or Y; +/UAS-GlcAT-P-RNAi; byn-Gal4, UAS-GFP, tub-Gal80*^{TS}*/+* (a),*+/+ or Y; UAS-Ras*^{G12V}*, Apc-RNAi, UAS-P53-RNAi/+; byn-Gal4, UAS-GFP, tub-Gal80*^{TS}*/+* (c), *UAS-Arm*^{CA}*/+ or Y; +/+; byn-Gal4, UAS-GFP, tub-Gal80*^{TS}*/+* (b).

Supplementary Figure 3:
VilCreER^{T2} *Apc*$^{fl/fl}$, *Kras*$^{G12D/+}$, *Trp53*$^{fl/fl}$ (a-h).

Supplementary Figure 4:
+/+ or Y; UAS-GFP/+; byn-Gal4, UAS-GFP, tub-Gal80^{TS}*/+* (a), *+/+ or Y; UAS-Ras*^{G12V}*/+; byn-Gal4, UAS-GFP, tub-Gal80*^{TS}*/+* (a), *+/+ or Y; UAS-Ras*^{G12V}*, Apc-RNAi, UAS-P53-RNAi/+; byn-Gal4, UAS-GFP, tub-Gal80*^{TS}*/+* (a, c and d), *+/+ or Y; UAS-Ras*^{G12V}*/UAS-GFP; byn-Gal4, UAS-GFP, tub-Gal80*^{TS}*/+* (b), *+/+ or Y; UAS-Ras*^{G12V}*/UAS-GFP; byn-Gal4, UAS-GFP, tub-Gal80*^{TS}*/UAS-nej-RNAi* (b).
